# Multi dimensional variable influence mechanism analysis for wheat biomass estimation using fused UAV spectral and canopy height data and machine learning

**DOI:** 10.3389/fpls.2026.1887967

**Published:** 2026-07-21

**Authors:** Shaoshuai Zhao, Shanjun Luo, Zhice Fang, Xiangchen Liu, Jiguang Chen

**Affiliations:** 1 College of Geographical Sciences, Faculty of Geographical Science and Engineering, Henan University, Zhengzhou, China; 2 Aerospace Information Research Institute, Henan Academy of Sciences, Zhengzhou, China; 3 School of Electronics and Information, Zhengzhou University of Aeronautics, Zhengzhou, China

**Keywords:** biomass, interpretability analysis, machine learning, remote sensing, UAV, wheat

## Abstract

**Introduction:**

Accurate and non-destructive estimation of wheat biomass is essential for crop growth monitoring, yield prediction, and precision agriculture. Unmanned aerial vehicle (UAV)-based remote sensing, integrating both spectral and structural information, has shown great potential for biomass estimation. However, the mechanisms by which different types of variables contribute to biomass prediction remain poorly understood, especially when using machine learning models.

**Methods:**

In this study, we fused spectral reflectance, vegetation indices, and canopy height data derived from a UAV multispectral camera to estimate wheat biomass across four growth stages (jointing, booting, heading, and filling). Four machine learning algorithms—XGBoost, Random Forest Regressor (RFR), Support Vector Regressor (SVR), and LASSO—were employed and compared.

**Results and discussion:**

The results showed that XGBoost achieved the highest accuracy (R^2^ = 0.919, RMSE = 102.43 g/m², MAE = 77.43 g/m², RRMSE = 19.71%). Furthermore, SHAP (SHapley Additive exPlanations) analysis revealed that canopy height (CH) was the most important variable, followed by spectral indices such as R842 and GNDVI. The univariate and global contribution analyses demonstrated that structural and spectral variables played complementary roles in biomass estimation. This study provides a mechanistic understanding of variable contributions and offers a robust framework for UAV-based wheat biomass estimation.

## 1 Introduction

Wheat (Triticum aestivum L.) is one of the most important staple crops worldwide, providing approximately 20% of the calories and protein consumed by the global population (Shiferaw et al., 2013). With the increasing demand for food security under climate change and resource constraints, precise monitoring of wheat growth and biomass accumulation has become a critical task for agricultural management and yield prediction (Liu et al., 2024). Biomass, defined as the total dry matter accumulation per unit area, is a key indicator of crop health, photosynthetic efficiency, and final grain yield (Zhu et al., 2023). Therefore, accurate and timely estimation of wheat biomass is essential for optimizing irrigation, fertilization, and pest control strategies, as well as for predicting yield at regional and global scales (Duan et al., 2026).

Traditional methods for biomass estimation rely on destructive sampling, which involves harvesting, drying, and weighing plant samples from representative plots. Although this approach provides accurate ground-truth data, it is labor-intensive, time-consuming, and spatially limited (Wei et al., 2023). Moreover, destructive sampling cannot be repeated on the same plot over time, making it unsuitable for continuous monitoring of crop growth dynamics (Li et al., 2024). To overcome these limitations, remote sensing technologies have been widely adopted for non-destructive biomass estimation (Smith et al., 2024).

Satellite-based remote sensing, such as data from Sentinel-2, Landsat, and MODIS, has been used for large-scale biomass mapping (Swoish et al., 2022; Wang et al., 2026). However, satellite imagery often suffers from coarse spatial resolution, cloud interference, and long revisit intervals, which limit its applicability for fine-scale field monitoring (Liu et al., 2025). In contrast, unmanned aerial vehicle (UAV)-based remote sensing offers several advantages, including ultra-high spatial resolution (centimeter-level), flexible flight scheduling, and the ability to carry various sensors (Meng et al., 2025). As a result, UAVs have become an increasingly popular platform for precision agriculture research (Zhang et al., 2025).

Recent studies have demonstrated that UAV-based multispectral imagery can provide valuable spectral information for biomass estimation (Singh et al., 2025). Key spectral features include canopy reflectance in the visible and near-infrared (NIR) bands, as well as vegetation indices such as the normalized difference vegetation index (NDVI), green NDVI (GNDVI), and the enhanced vegetation index (EVI) (Hu et al., 2025). These spectral variables are sensitive to canopy chlorophyll content, leaf area index (LAI), and canopy structure, all of which are closely related to biomass (Singh et al., 2025).

In addition to spectral data, crop canopy structure—such as canopy height (CH)—has been recognized as an important predictor of biomass (Liu et al., 2025). Canopy height is directly related to plant growth stage, stem elongation, and lodging, and it provides complementary information that spectral data alone cannot capture. With the development of structure-from-motion (SfM) photogrammetry, UAV-based point clouds can be generated from overlapping images, allowing the extraction of high-resolution canopy height models (CHMs) (Li et al., 2025). The integration of spectral and structural data has been shown to improve biomass estimation accuracy compared to using either data type alone (Yang et al., 2026; Meng et al., 2025).

However, despite the growing body of research on UAV-based biomass estimation, several critical gaps remain. First, most studies have focused on evaluating the overall performance of machine learning models (e.g., Random Forest, Support Vector Machine, XGBoost) for biomass prediction, but few have systematically investigated how different types of variables—spectral reflectance, vegetation indices, and canopy height—contribute to the model’s prediction mechanism (Yao et al., 2026). Second, machine learning models are often treated as “black boxes,” making it difficult to interpret which variables are most influential and why (Xu et al., 2026). This lack of interpretability limits the scientific understanding of the underlying biophysical processes and the practical guidance for feature selection in future studies. Third, the influence of different variables may vary across growth stages and biomass levels, yet such temporal and dynamic effects have rarely been analyzed (Su et al., 2025).

To address these gaps, this study aims to: (1) integrate UAV-derived spectral features (reflectance and vegetation indices) and structural features (canopy height) to estimate wheat biomass across four key growth stages; (2) compare the performance of four machine learning algorithms (XGBoost, RFR, SVR, and LASSO) in biomass estimation; (3) use SHAP (SHapley Additive exPlanations) analysis to quantify the global and local contributions of each variable; and (4) analyze the variable influence mechanisms, including univariate response patterns and differences across biomass levels. This study provides a comprehensive interpretability framework for UAV-based biomass estimation and offers practical insights for precision wheat management.

## 2 Materials and methods

### 2.1 Experimental design

**Figure 1:**
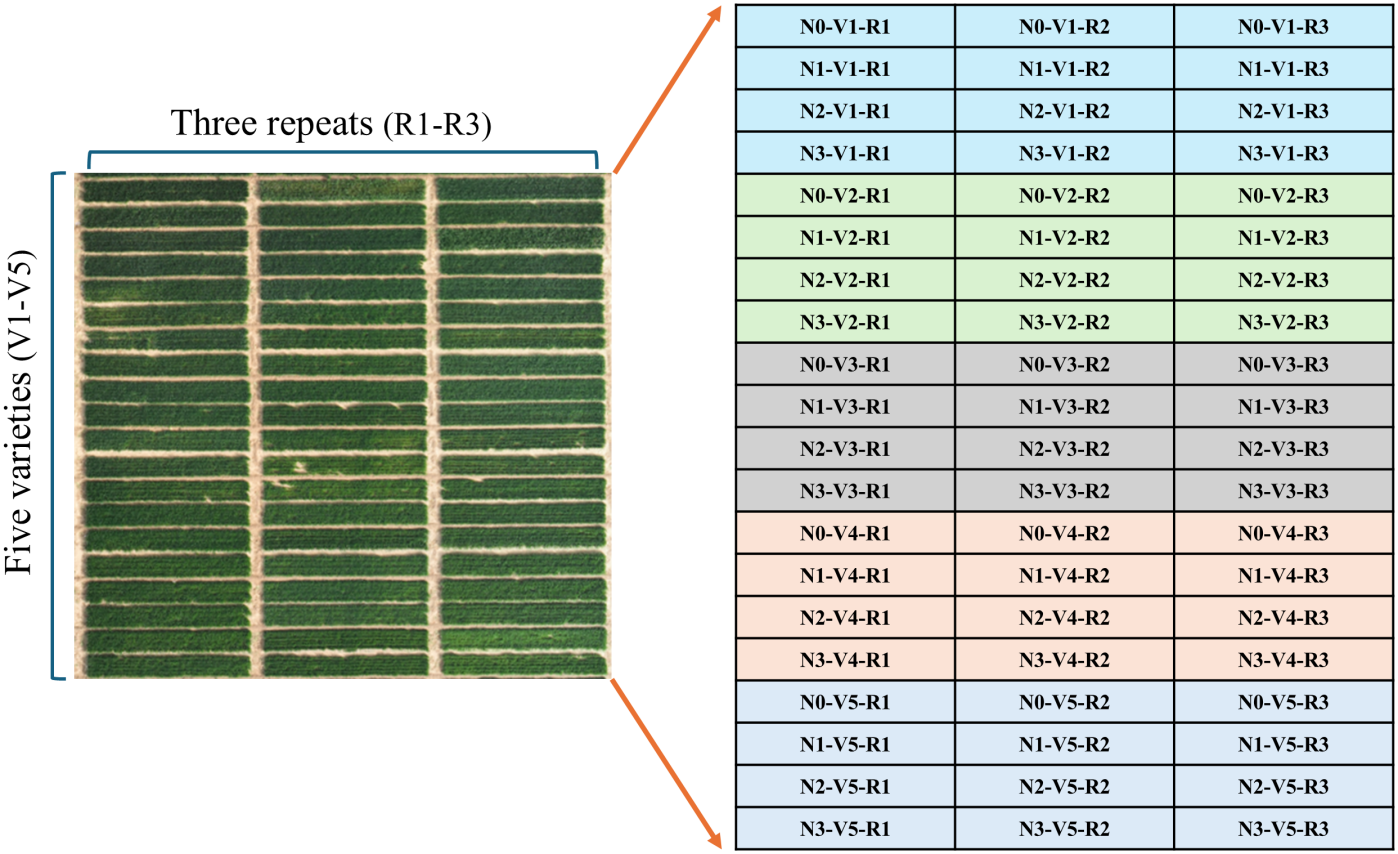
Schematic layout of the field experimental plots.

The field experiment was conducted in Jiaozuo City, Henan Province, China. Five locally representative winter wheat varieties were selected for the study, with three replicates, resulting in a total of 60 experimental plots. Each plot had an area of approximately 15 m² (1.5 m × 10 m), with a 0.5 m buffer zone between adjacent plots to minimize edge effects and nutrient interference. The layout of the plots is shown in **Figure 1**.

Four nitrogen (N) fertilization levels were applied: N0 (0 kg N/ha), N1 (100 kg N/ha), N2 (200 kg N/ha), and N3 (300 kg N/ha). Nitrogen fertilizer was applied in the form of urea (46% N), with 60% applied as basal fertilizer before sowing and the remaining 40% applied as topdressing in two split applications: 20% at the jointing stage and 20% at the heading stage. Phosphorus (P_2_O_5_, 90 kg/ha) and potassium (K_2_O, 120 kg/ha) were applied as basal fertilizers uniformly across all plots to ensure non-limiting conditions for P and K.

All other agronomic management practices, including irrigation, weeding, and pest and disease control, were carried out according to local conventional management protocols to reflect typical field conditions in the Jiaozuo region. No significant lodging or severe stress was observed during the growing season.

### 2.2 UAV multispectral image acquisition and processing

Multispectral images were acquired using a RedEdge-P camera (Micasense Co., Ltd., Seattle, Washington, USA) mounted on a DJI Matrice 350 unmanned aerial vehicle (UAV). The camera captured five spectral bands: blue (475 nm), green (560 nm), red (668 nm), red edge (717 nm), and near-infrared (842 nm). UAV flights were conducted at four key wheat growth stages: jointing (March 25, 2025), booting (April 15, 2025), heading (April 23, 2025), and filling stages (May 20, 2025).

Before each flight, the flight route was planned using the DJI Pilot 2 software. The flight altitude was set to 40 m above ground level, resulting in a ground sampling distance (GSD) of approximately 20 mm. The flight speed was maintained at 5 m/s, with an 80% forward overlap and 70% side overlap to ensure sufficient image coverage for three-dimensional reconstruction. The flight path was designed to cover the entire experimental area, and each flight was conducted under clear sky conditions between 10:00 and 14:00 local time to minimize solar angle effects.

A calibrated reflectance panel (Micasense Calibrated Reflectance Panel, 50% reflectance) was used for radiometric calibration. The panel was placed on the ground before and after each flight, and images of the panel were captured for subsequent reflectance correction. The panel was positioned horizontally and perpendicular to the sunlight to ensure accurate reflectance measurements.

All acquired images were processed using Agisoft Metashape Professional (Version 2.1.0, Agisoft LLC Co., Ltd., Russia). The processing workflow included the following steps:

1. Image import and quality assessment: Images with motion blur or poor illumination were removed.
2. Radiometric calibration: The images were calibrated using the reflectance panel images to convert digital numbers (DN) to reflectance values.
3. Image alignment: Tie points were generated using the SIFT algorithm, and the images were aligned with high accuracy settings.
4. Dense cloud generation: Dense point clouds were generated using the depth maps method.
5. Digital surface model (DSM) generation: The DSM was generated based on the dense point cloud.
6. Orthomosaic generation: The orthomosaic was exported from the DSM.

### 2.3 Wheat biomass acquisition

Destructive sampling was conducted to obtain the ground-truth wheat biomass for each plot. In each of the 60 experimental plots, consistent with the acquisition time of the UAV images, a representative sampling area of 0.2 m × 0.2 m (0.04 m²) was randomly selected, avoiding the border rows to eliminate edge effects. All wheat plants within the sampling area were cut at ground level using scissors. The collected plant samples were immediately placed in labeled paper bags and transported to the laboratory for further processing. In the laboratory, the samples were oven-dried at 85 °C for 48 hours until a constant weight was achieved. The dry weight of each sample was recorded using an electronic balance with a precision of 0.01 g. To obtain the biomass per unit area (g/m²) for each plot, the dry weight (g) was converted using the following formula (Equation 1):

(1) $$\begin{array}{c} Biomass\;(\frac{g}{m^{2}})=\frac{Dry\;weight\;(g)}{0.04\;m^{2}} \end{array}$$

This conversion yielded the wheat biomass in g/m² for each plot at each sampling stage. A total of 240 biomass samples (60 plots × 4 growth stages) were collected and processed for subsequent analysis.

### 2.4 Spectral information extraction

For each plot, spectral information was extracted from the orthomosaic generated from the UAV multispectral images. To avoid edge effects, a region of interest (ROI) was defined within each plot by removing the border area (approximately 0.5 m from the plot edges). The mean spectral reflectance of all pixels within the ROI was then calculated for each of the five spectral bands (blue, green, red, red edge, and near-infrared). This mean reflectance value was used as the canopy spectral reflectance for that plot.

Based on the extracted canopy reflectance, eight vegetation indices were calculated to capture spectral characteristics related to crop growth and biomass accumulation. In total, the spectral information used in this study consisted of the five band reflectance values and the eight vegetation indices, resulting in 13 spectral features for each plot. The formulas for each vegetation index are listed in **Table 1**.

**Table 1:** Vegetation indices used in this study.

Vegetation index	Formula	Reference
NDVI	(NIR – Red)/(NIR + Red)	(Rouse et al., 1974)
GNDVI	(NIR – Green)/(NIR + Green)	(Gitelson and Merzlyak, 1996)
EVI	2.5 × ((NIR – Red)/(NIR + 6 × Red – 7.5 × Blue + 1))	(Shen et al., 2024)
NDRE	(NIR – Red Edge)/(NIR + Red Edge)	(Gitelson and Merzlyak, 1994)
SAVI	((NIR – Red)/(NIR + Red + L)) × (1 + L), with L = 0.5	(Killeen et al., 2024)
OSAVI	(NIR – Red)/(NIR + Red + 0.16)	(Killeen et al., 2024)
CIre	(NIR/Red Edge) – 1	(Gitelson et al., 2003)
SR	NIR/Red	(Yang et al., 2024)

### 2.5 Extraction of canopy structure information

Canopy structure information was extracted in the form of canopy height model (CHM), which represents the vertical distribution of crop canopy above the ground surface (Liu et al., 2025; Lu et al., 2019). To obtain the CHM for each growth stage, a digital elevation model (DEM) and digital surface models (DSMs) were generated from the UAV imagery. The DEM was generated from UAV images acquired before crop emergence, when the field surface was bare soil with no vegetation cover. The DEM represented the bare ground elevation and served as the baseline reference for canopy height calculation. The DSM for each growth stage (jointing, booting, heading, and filling) was generated from the orthomosaic processing workflow described in Section 2.2. For each growth stage, the CHM was calculated by subtracting the DEM from the corresponding DSM using the following formula (Equation 2):

(2) $$\begin{array}{c} CHM=DSM-DEM \end{array}$$

The CHM values represented the absolute canopy height (m) above the ground surface. For each plot, the mean canopy height was calculated by extracting the CHM values within the same ROI defined in Section 2.4. The mean canopy height for each plot at each growth stage was used as the structural feature for subsequent analysis. This CHM-based method effectively captures the vertical growth characteristics of wheat canopies, providing complementary structural information to the spectral data.

### 2.6 Machine learning methods

In this study, four machine learning algorithms were selected for wheat biomass estimation: XGBoost, Random Forest Regressor (RFR), Support Vector Regressor (SVR), and LASSO (Least Absolute Shrinkage and Selection Operator). These algorithms were chosen for the following reasons: (1) they represent different paradigms of machine learning, including ensemble learning (XGBoost and RFR), kernel-based methods (SVR), and regularized linear regression (LASSO); (2) they have been widely used in remote sensing-based crop parameter estimation; and (3) they offer different levels of interpretability and computational efficiency, allowing for a comprehensive comparison of model performance.

XGBoost (Extreme Gradient Boosting) is a scalable and efficient gradient boosting framework that builds an ensemble of decision trees sequentially (Amazirh et al., 2026). Unlike traditional gradient boosting, XGBoost incorporates regularization terms to prevent overfitting and uses a sparsity-aware algorithm to handle missing values. It also supports parallel processing and tree pruning, making it suitable for large-scale datasets with complex non-linear relationships. XGBoost has been shown to perform well in various agricultural remote sensing applications (Sánchez et al., 2025; Bourbour et al., 2025).

Random Forest Regressor (RFR) is an ensemble learning method that constructs multiple independent decision trees using bootstrap samples of the training data (Kheir et al., 2022). The final prediction is obtained by averaging the outputs of all individual trees. RFR is robust to outliers and noise, can handle high-dimensional data, and provides inherent feature importance scores. Its ability to capture non-linear interactions without extensive hyperparameter tuning makes it a popular choice for crop parameter estimation (Xiao et al., 2026).

Support Vector Regressor (SVR) is a kernel-based method that maps input data into a high-dimensional feature space and finds a hyperplane that best fits the data while minimizing prediction error within a specified margin (Shafiee et al., 2021). SVR is effective for small to medium-sized datasets and can handle non-linear relationships using different kernel functions (e.g., radial basis function, linear, and polynomial). Its generalization ability is often superior to linear models when dealing with complex patterns (Keselj et al., 2025).

LASSO (Least Absolute Shrinkage and Selection Operator) is a linear regression method with L1 regularization, which penalizes the absolute values of regression coefficients (Haseeb et al., 2025). This penalty forces some coefficients to become exactly zero, effectively performing feature selection. LASSO is particularly useful when the number of features is large relative to the number of samples, as it helps reduce overfitting and improve model interpretability by selecting only the most relevant variables (Kumar et al., 2019).

### 2.7 SHAP method

To interpret the output of the machine learning models and quantify the contribution of each input feature to wheat biomass estimation, the SHAP (SHapley Additive exPlanations) method was employed in this study. SHAP is a unified framework for interpreting model predictions based on cooperative game theory, specifically the Shapley values, which assign an importance value to each feature for a given prediction (Zhong et al., 2026).

The SHAP method calculates the marginal contribution of each feature by considering all possible subsets of features and averaging their contributions. The fundamental formula for the SHAP value of a feature *i* (*ϕ_i_*) for a specific prediction *x* is defined as Equation 3:

(3) $$\begin{array}{c} \phi_{i(x)}=\sum_{S\;\subseteq F\setminus \{i\}}\;\frac{|S|!(|F|-|S|-1)!}{|F|!}[f(S\cup \{i\})-f(S)] \end{array}$$

Where *F* is the set of all features, *S* is a subset of features excluding feature *i*, and *f*(*S*) is the model prediction using only the features in subset *S*. The SHAP value $\;\phi_{i}(x)$ represents the average marginal contribution of feature *i* to the prediction for sample *x*.

In this study, SHAP analysis was performed on the optimal model, which achieved the highest prediction accuracy among the four machine learning algorithms. The analysis was conducted from both global and local interpretability perspectives. Global interpretability was achieved by computing the mean absolute SHAP value for each feature across all samples, providing a ranking of overall feature importance. Local interpretability was achieved by examining the SHAP value distribution for individual predictions, revealing how specific feature values contributed to each biomass estimate. This dual-level interpretation framework has been increasingly adopted in remote sensing studies to bridge the gap between model performance and mechanistic understanding of vegetation growth dynamics (Liu et al., 2025; Yuan et al., 2024). The following SHAP-based visualizations were generated:

1. SHAP summary plot (bar chart): This plot shows the global feature importance by displaying the mean absolute SHAP value for each feature across all samples. Features are ranked in descending order of importance.
2. SHAP summary plot (beeswarm): This plot displays the distribution of SHAP values for each feature across all samples. Each dot represents a single sample, with the color indicating the feature value (red for high, blue for low) and the x-axis position representing the SHAP value (positive or negative impact on model output).
3. SHAP dependence plots: These scatter plots show the relationship between a single feature and its SHAP values, revealing non-linear effects and potential interactions with other features.

The SHAP analysis provides a transparent and interpretable explanation of how each variable contributes to the biomass prediction, which is essential for understanding the underlying biophysical mechanisms and guiding feature selection in future studies. The SHAP calculations and visualizations were implemented using the Python SHAP library (Version 0.41.0).

### 2.8 Model performance evaluation

When using machine learning or deep learning, randomly dividing the dataset into training and testing sets in a ratio of approximately 8:2 or 7:3 is a widely accepted and reasonable partitioning strategy in the field of biomass remote sensing estimation (Wang et al., 2016). In this study, the entire dataset was randomly split into a training set and a validation set at a ratio of 8:2, with 80% of the samples used for model training and the remaining 20% reserved for independent validation. This split ensures that the model performance is evaluated on unseen data, reducing the risk of overfitting. Four statistical metrics were used to evaluate the performance of the machine learning models: coefficient of determination (R^2^), root mean square error (RMSE), mean absolute error (MAE), and relative root mean square error (RRMSE). Higher R*^2^* values indicate better model fit, while lower RMSE, MAE, and RRMSE values indicate better prediction accuracy.

Hyperparameter tuning was performed using grid search with 5-fold cross-validation to optimize model performance for each machine learning algorithm. The optimal hyperparameters were selected based on the lowest RMSE achieved during cross-validation. All model training and evaluation procedures were implemented using Python (Version 3.9) with the scikit-learn library.

## 3 Results

### 3.1 Statistical summary of wheat biomass samples

A statistical summary of the measured wheat aboveground biomass (AGB) across all 240 samples is presented in **Table 2**. Across the entire dataset, AGB ranged from 38.78 to 1230.89 g/m², with a mean value of 486.86 g/m² and a standard deviation (SD) of 329.00 g/m². The coefficient of variation (CV) was 67.58%, indicating substantial variability across different growth stages, nitrogen treatments, and wheat varieties, which is beneficial for developing robust machine learning models.

**Table 2:** Statistical information of wheat biomass samples.

Statistical indices	All data	Training data	Test data
Maximum (g/m^2^)	1230.89	1230.89	1146.03
Minimum (g/m^2^)	38.78	38.78	50.68
Mean (g/m^2^)	486.86	478.67	519.60
Standard deviation (SD) (g/m^2^)	329.00	325.80	339.54
Coefficient of variation (CV) (%)	67.58	68.06	65.35

To evaluate the representativeness of the data split, we also compared the statistical distributions of the training set (80% of samples, n = 192) and the test set (20% of samples, n = 48). The training set exhibited a mean of 478.67 g/m² (SD = 325.80 g/m², CV = 68.06%), with values ranging from 38.78 to 1230.89 g/m². The test set showed a mean of 519.60 g/m² (SD = 339.54 g/m², CV = 65.35%), with values ranging from 50.68 to 1146.03 g/m². The comparable means, standard deviations, and coefficients of variation between the two subsets confirm that the random 8:2 split produced training and test sets with similar statistical properties, ensuring that the test set is representative of the overall dataset and that the model evaluation is unbiased.

### 3.2 Correlation analysis between single indicator and biomass

**Figure 2:**
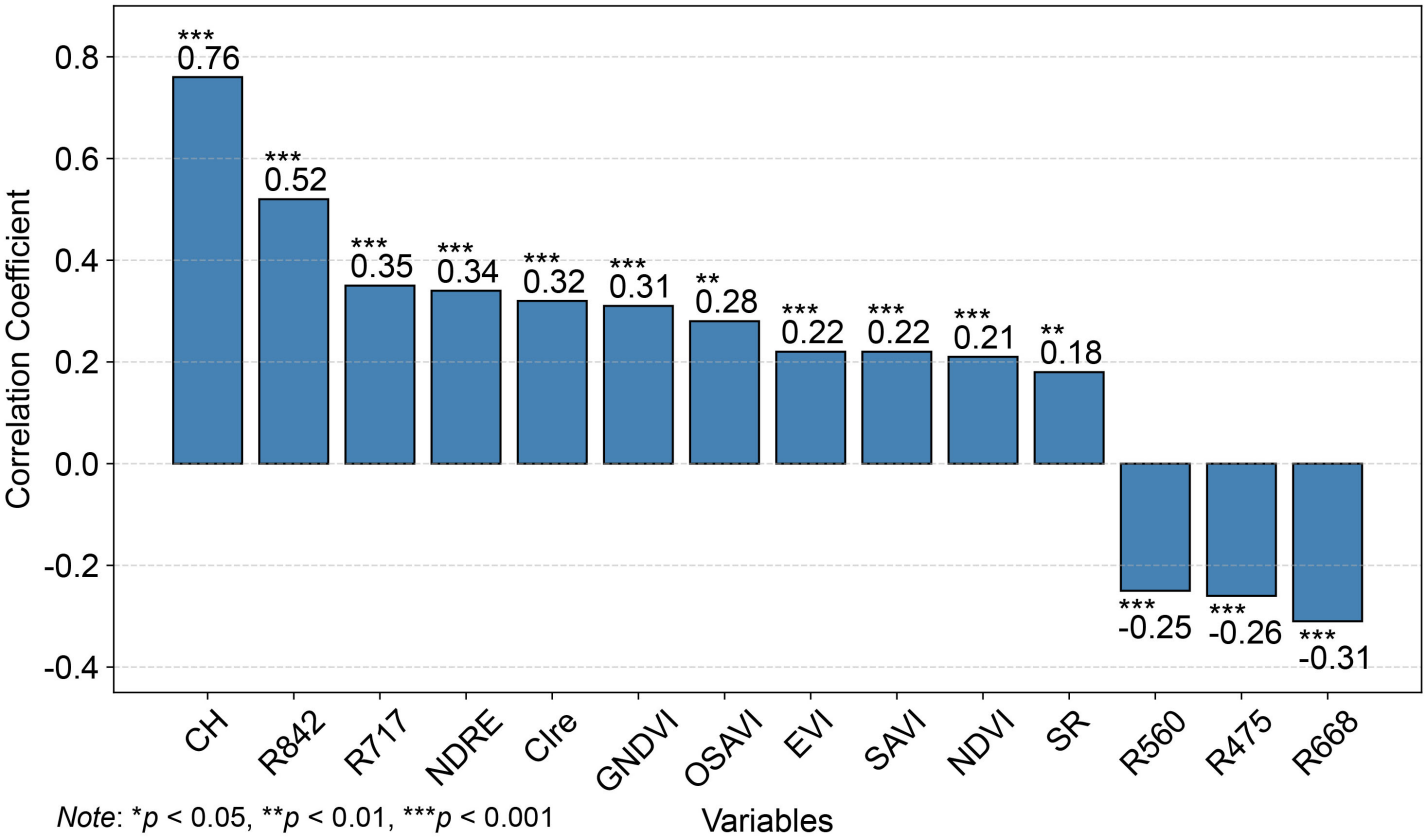
Correlation analysis between individual spectral/structural variables and wheat aboveground biomass.

To preliminarily assess the relationship between individual input variables and wheat aboveground biomass (AGB), Pearson correlation analysis was conducted between each spectral and structural feature and the measured biomass values. Correlation coefficients were calculated based on pooled samples from all four growth stages. The results are presented in **Figure 2**. Among all variables, canopy height (CH) exhibited the strongest positive correlation with biomass, with a correlation coefficient of *r* = 0.76. This strong positive correlation indicates that plant height is a reliable indicator of aboveground biomass accumulation, consistent with the biological understanding that taller canopies typically have greater stem elongation and dry matter accumulation. The near-infrared reflectance band (R842) ranked second with a correlation coefficient of *r* = 0.52. The high sensitivity of NIR reflectance to canopy structure and leaf internal scattering makes it a valuable spectral predictor of biomass. The red edge band (R717) and the normalized difference red edge index (NDRE) showed moderate positive correlations of *r* = 0.35 and *r* = 0.34, respectively. These features are known to be sensitive to canopy chlorophyll content and nitrogen status, which are closely linked to photosynthetic capacity and biomass accumulation.

Among the vegetation indices, CIre and GNDVI exhibited moderate positive correlations with biomass, with coefficients of *r* = 0.32 and *r* = 0.31, respectively. OSAVI and EVI showed slightly lower positive correlations of *r* = 0.28 and *r* = 0.22, while SAVI also displayed a correlation of *r* = 0.22. The normalized difference vegetation index (NDVI) and the simple ratio index (SR) showed the lowest positive correlations among the vegetation indices, with coefficients of *r* = 0.21 and *r* = 0.18, respectively. In contrast, the three visible reflectance bands exhibited negative correlations with biomass. The blue band (R475) showed a correlation of *r* = −0.26, the green band (R560) showed a correlation of *r* = −0.25, and the red band (R668) exhibited the strongest negative correlation of *r* = −0.31. These negative correlations suggest that higher reflectance in the visible bands (indicating lower pigment absorption) is associated with lower biomass, which is biologically reasonable as healthy, high-biomass canopies typically exhibit strong absorption in the blue and red regions due to chlorophyll pigments.

Overall, the correlation analysis revealed that structural information (CH) was most strongly related to biomass, followed by NIR reflectance (R842) and red edge-related features (R717 and NDRE). The negative correlations observed for the visible reflectance bands further confirm the importance of pigment absorption for biomass estimation. These findings provide a basis for subsequent machine learning modeling and variable importance analysis.

### 3.3 Performance comparison of different machine learning models for wheat biomass estimation

**Figure 3:**
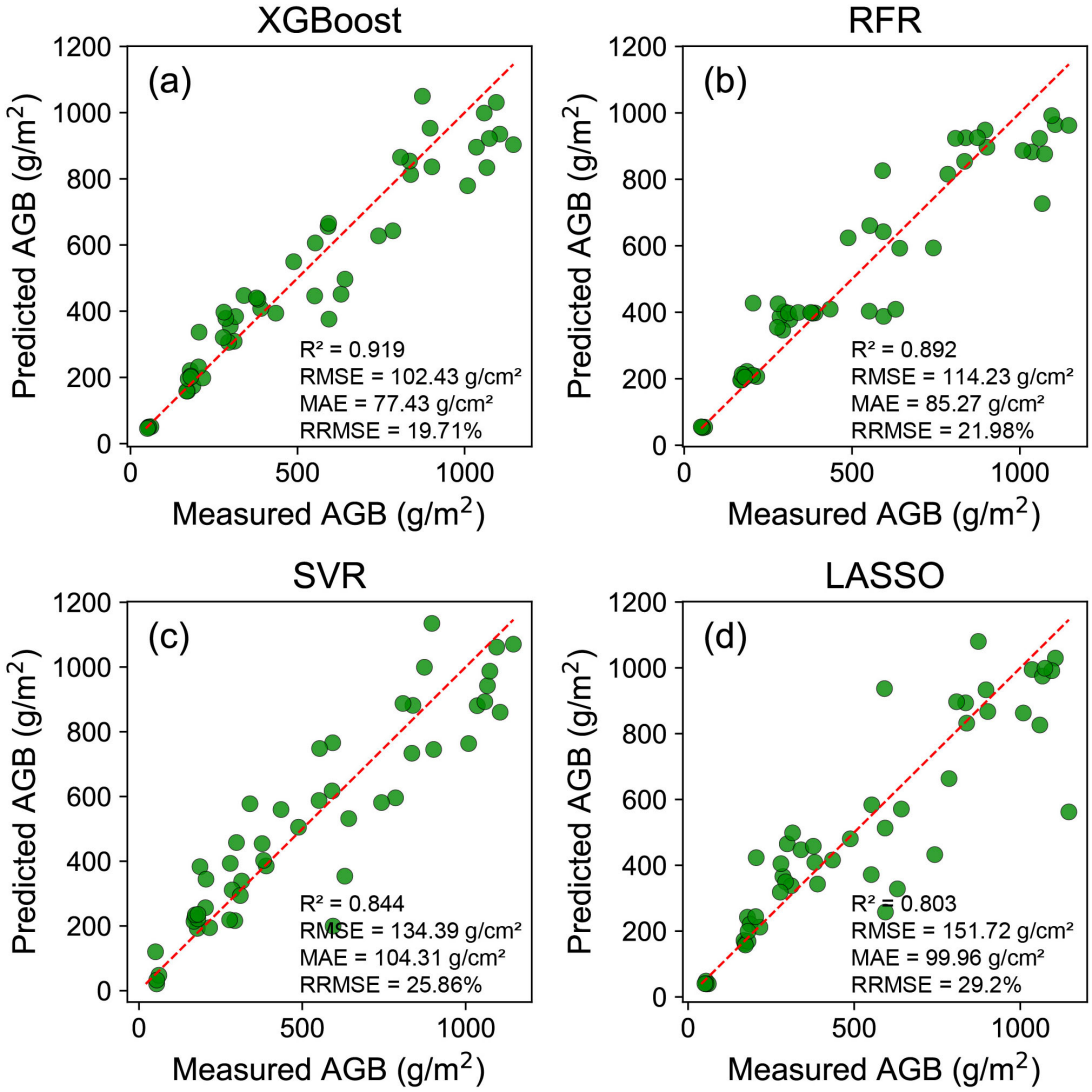
Comparison of measured and predicted wheat biomass using different machine learning models. **(a)** XGBoost, **(b)** RFR, **(c)** SVR, **(d)** LASSO.

The performance of four machine learning models (XGBoost, RFR, SVR, and LASSO) for estimating wheat aboveground biomass (AGB) was evaluated using the validation dataset. The hyperparameter settings for machine learning are shown in **Table 3**. The scatter plots of measured versus predicted AGB for each model are shown in **Figure 3**.

**Table 3:** Main hyperparameter settings for machine learning.

Model	Parameter	Search space
XGBoost	Number of boosting rounds	randint (100, 1500)
	Learning rate	uniform (0.01, 0.5)
	Maximum tree depth	randint (3, 10)
	Subsample ratio of the training instance	uniform (0.4, 0.6)
RFR	Number of boosting rounds	randint (100, 1500)
	Maximum tree depth	randint (3, 10)
	Minimum number of samples for each split	randint (3, 10)
	Minimum number of samples for each node	randint (3, 10)
SVR	Kernel	[rbf, linear, poly]
	C	[0.1, 1, 10, 100]
	gamma	[0.001, 0.01, 0.1, 1, 10]
LASSO	alpha	[0.001, 0.005, 0.01, 0.05, 0.1, 0.5, 1]

XGBoost achieved the highest estimation accuracy among the four models, with an R^2^ of 0.919, RMSE of 102.43 g/m², MAE of 77.43 g/m², and RRMSE of 19.71% (**Figure 3a**). The data points were tightly clustered along the 1:1 line, indicating strong agreement between measured and predicted values. The relatively low RMSE and MAE values further demonstrated that XGBoost had the smallest prediction error. Random Forest Regressor (RFR) ranked second with an R^2^ of 0.892, RMSE of 114.23 g/m², MAE of 85.27 g/m², and RRMSE of 21.98% (**Figure 3b**). Although RFR performed slightly worse than XGBoost, it still exhibited satisfactory predictive capability and reasonable generalization performance. Support Vector Regressor (SVR) showed moderate performance, with an R^2^ of 0.844, RMSE of 134.39 g/m², MAE of 104.31 g/m², and RRMSE of 25.86% (**Figure 3c**). The scatter points showed greater dispersion around the 1:1 line compared to XGBoost and RFR, particularly at higher biomass levels, suggesting that SVR was less effective in capturing non-linear relationships in the dataset. LASSO exhibited the lowest accuracy among the four models, with an R^2^ of 0.803, RMSE of 151.72 g/m², MAE of 99.96 g/m², and RRMSE of 29.20% (**Figure 3d**). The higher RMSE and RRMSE values indicated that LASSO, as a linear model with L1 regularization, struggled to capture the complex non-linear relationships between spectral-structural features and wheat biomass.

Overall, the ensemble learning methods (XGBoost and RFR) outperformed SVR and LASSO in estimating wheat biomass. The superior performance of XGBoost can be attributed to its ability to handle non-linear interactions, incorporate regularization to prevent overfitting, and efficiently utilize both spectral and structural features. These results suggest that XGBoost is the most suitable machine learning algorithm for UAV-based wheat biomass estimation in this study.

### 3.4 Performance comparison of different variable fusion schemes for wheat biomass estimation

**Figure 4:**
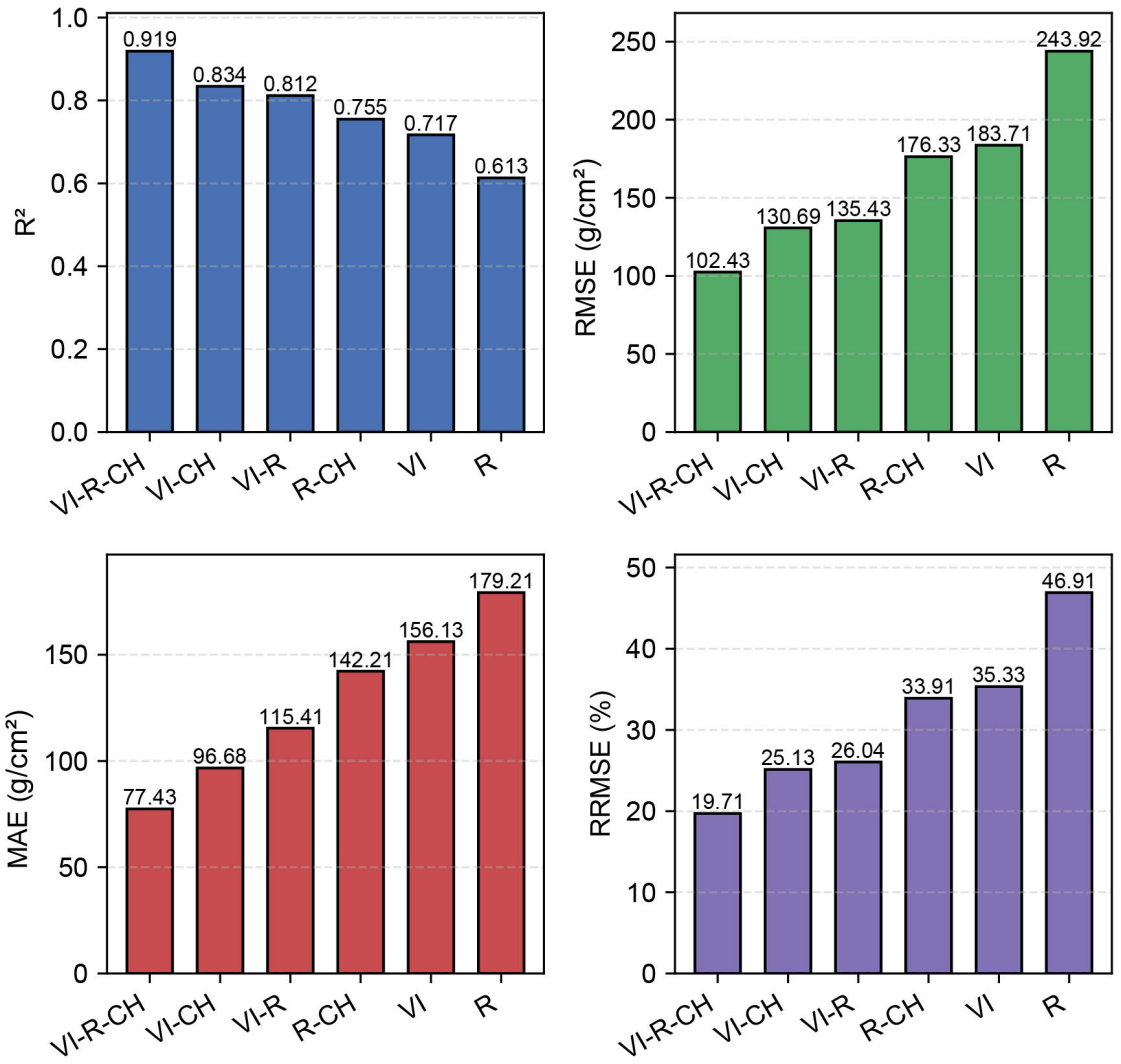
Performance comparison of different variable fusion schemes for wheat biomass estimation.

To evaluate the effectiveness of different variable combinations for wheat biomass estimation, six input schemes were compared using the optimal XGBoost model: (1) VI-R-CH: fusion of vegetation indices, spectral reflectance, and canopy height; (2) VI-CH: fusion of vegetation indices and canopy height; (3) VI-R: fusion of vegetation indices and spectral reflectance; (4) R-CH: fusion of spectral reflectance and canopy height; (5) VI: vegetation indices only; and (6) R: spectral reflectance only. The performance metrics (R^2^, RMSE, MAE, and RRMSE) for each scheme are presented in **Figure 4**.

The VI-R-CH scheme, which integrated all three types of features, achieved the highest estimation accuracy with an R^2^ of 0.919, RMSE of 102.43 g/m², MAE of 77.43 g/m², and RRMSE of 19.71%. This result demonstrates that the fusion of spectral reflectance, vegetation indices, and canopy height provides the most comprehensive information for wheat biomass estimation. The VI-CH scheme ranked second with an R^2^ of 0.834, RMSE of 130.69 g/m², MAE of 96.68 g/m², and RRMSE of 25.13%. Compared to the full fusion scheme, the removal of spectral reflectance led to a noticeable decrease in R^2^ (from 0.919 to 0.834) and an increase in RMSE (from 102.43 to 130.69 g/m²), highlighting the importance of spectral information for biomass estimation. The VI-R scheme showed comparable performance to VI-CH, with an R^2^ of 0.812, RMSE of 135.43 g/m², MAE of 115.41 g/m², and RRMSE of 26.04%. Although the R^2^ was slightly lower than VI-CH, the differences in RMSE and MAE were relatively small, indicating that spectral reflectance and vegetation indices provide complementary spectral information. The R-CH scheme achieved an R^2^ of 0.755, RMSE of 176.33 g/m², MAE of 142.21 g/m², and RRMSE of 33.91%. This scheme performed worse than both VI-CH and VI-R, suggesting that vegetation indices derived from spectral reflectance contain additional useful information beyond raw reflectance bands for biomass estimation. The VI and R schemes, which used only vegetation indices or only spectral reflectance, exhibited the lowest accuracies. The VI scheme achieved an R^2^ of 0.717, RMSE of 183.71 g/m², MAE of 156.13 g/m², and RRMSE of 35.33%. The R scheme performed the poorest with an R^2^ of 0.613, RMSE of 243.92 g/m², MAE of 179.21 g/m², and RRMSE of 46.91%. These results indicate that using either vegetation indices or spectral reflectance alone is insufficient for accurate biomass estimation.

In summary, the performance ranking of the six fusion schemes was: VI-R-CH > VI-CH > VI-R > R-CH > VI > R. The full fusion scheme (VI-R-CH) consistently outperformed all other combinations across all evaluation metrics. This confirms that integrating structural information (canopy height) with both spectral reflectance and vegetation indices significantly improves wheat biomass estimation accuracy. The results underscore the importance of multi-dimensional variable fusion for UAV-based crop monitoring.

**Figure 5:**
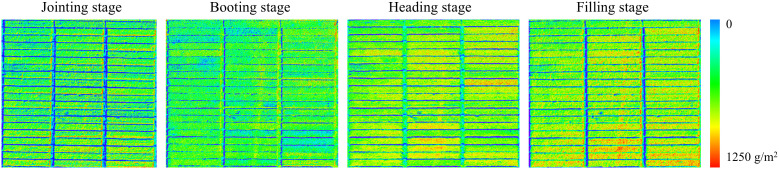
Spatial distribution mapping of wheat biomass prediction in different periods.

To further visualize the model’s performance across the growing season, we generated spatial distribution maps of predicted wheat AGB for the four growth stages using the optimal XGBoost model (**Figure 5**). The predicted biomass showed a clear increasing trend from the jointing stage to the filling stage, consistent with the expected pattern of wheat growth and biomass accumulation. At the jointing stage, the predicted AGB values were relatively low across all plots, reflecting the early vegetative growth phase. As the growing season progressed to the booting and heading stages, biomass increased substantially, with evident spatial variability among different plots due to differences in nitrogen treatments and wheat varieties. At the filling stage, the model predicted the highest biomass levels, corresponding to the peak of dry matter accumulation before senescence. Overall, the spatial distribution maps demonstrate that the XGBoost model effectively captures both the temporal progression and spatial heterogeneity of wheat biomass, further validating its reliability for field-scale biomass estimation.

### 3.5 Contribution mechanism analysis of different variables for wheat biomass estimation

#### 3.5.1 Global variable importance analysis

**Figure 6:**
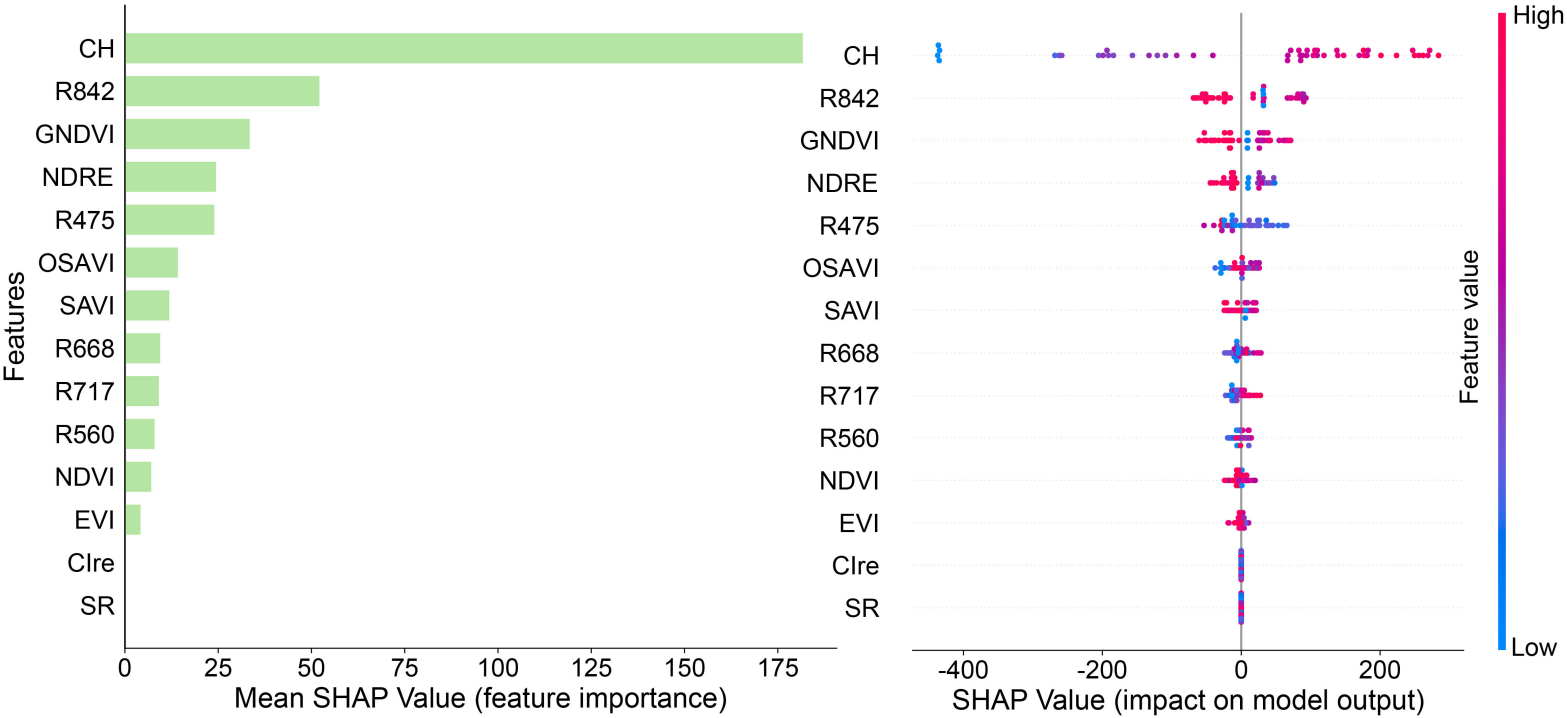
Global SHAP-based variable importance ranking (left) and contribution distribution (right).

To elucidate the contribution of each input feature to the wheat biomass estimation model, SHAP (SHapley Additive exPlanations) analysis was performed based on the optimal XGBoost model. The global feature importance, represented by the mean absolute SHAP values, is shown in **Figure 6** (left panel). Among all input variables, canopy height (CH) exhibited the highest mean SHAP value (approximately 175), indicating that it was the most important predictor for wheat biomass estimation. This result suggests that structural information plays a dominant role in determining aboveground biomass, which is consistent with the biological understanding that plant height is closely related to stem elongation, leaf area index, and overall dry matter accumulation.

Following CH, the near-infrared reflectance band (R842) ranked second with a mean SHAP value of approximately 50. This band is sensitive to canopy structural changes and internal leaf scattering, making it a reliable indicator of biomass. The green normalized difference vegetation index (GNDVI) and the normalized difference red edge index (NDRE) ranked third and fourth, respectively, with mean SHAP values of approximately 30 and 25. Both indices are commonly used to assess canopy chlorophyll content and nitrogen status, which are closely linked to photosynthetic capacity and biomass accumulation. The blue reflectance band (R475) showed moderate importance, while vegetation indices such as OSAVI, SAVI, R668, R717, and R560 exhibited lower mean SHAP values (ranging from 10 to 15). The lowest contributions were observed for NDVI, EVI, CIre, and SR, all with mean SHAP values below 10.

The SHAP summary plot (**Figure 6**, right panel) further reveals the direction and magnitude of each feature’s impact on model output. For CH, high feature values (indicated in red) were associated with positive SHAP values, while low feature values (blue) corresponded to negative SHAP values. This positive relationship confirms that taller canopies contribute to higher biomass predictions. Similarly, for R842 and GNDVI, high values generally resulted in positive contributions to biomass estimation, although some variability was observed due to interactions with other features. In contrast, for R475, the relationship was more complex: both high and low values were associated with positive and negative SHAP values, suggesting non-linear interactions with other variables. For lower-ranked features such as NDVI and EVI, the SHAP values were clustered near zero, indicating limited independent contributions to the model.

In summary, the global SHAP analysis revealed that structural information (CH) was the most influential variable, followed by spectral features (R842, GNDVI, and NDRE). These findings highlight the importance of integrating both structural and spectral data for accurate wheat biomass estimation.

#### 3.5.2 Univariate influence mechanism analysis

**Figure 7:**
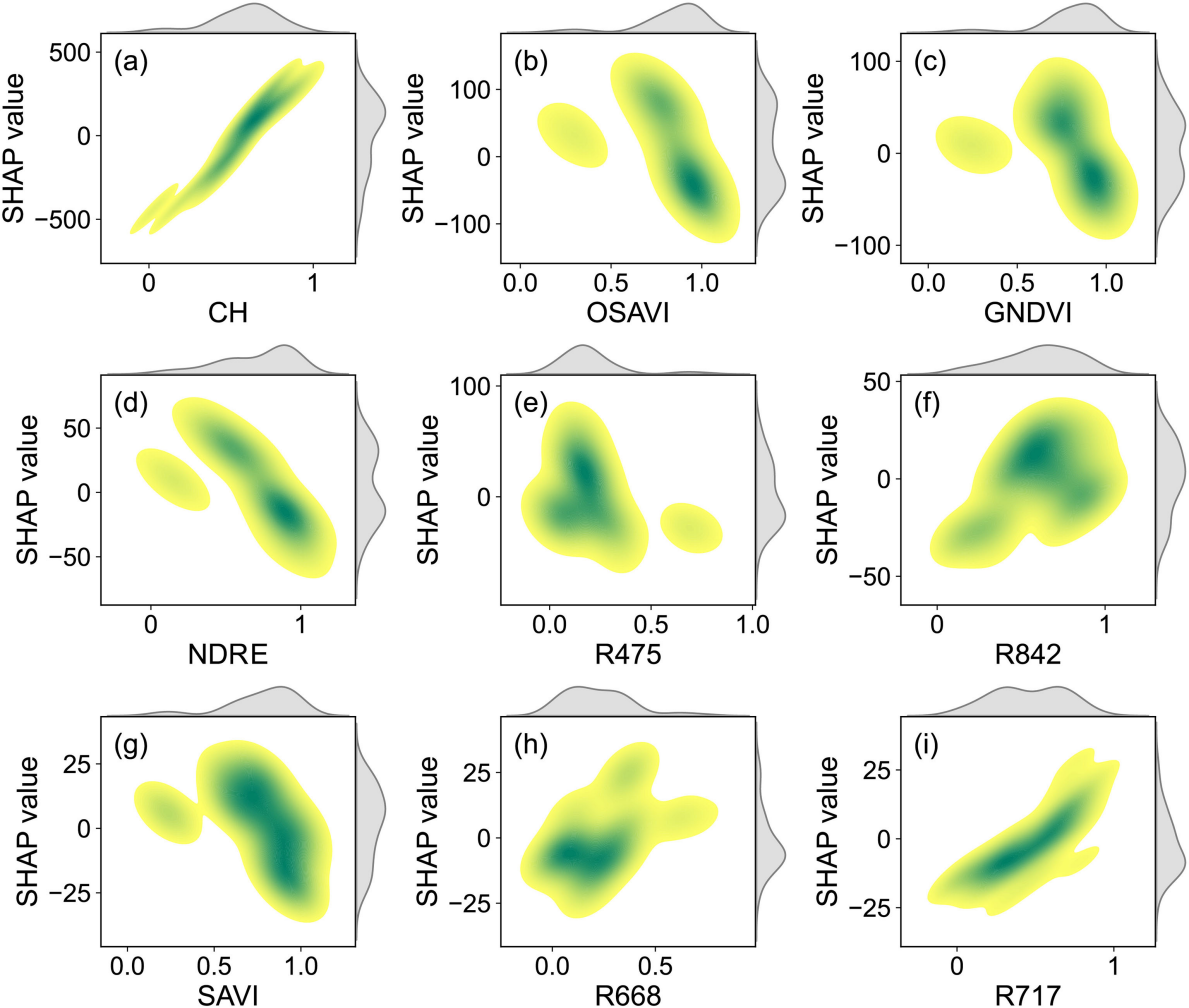
SHAP dependence plots for key variables: influence mechanisms of individual features on wheat biomass prediction. **(a)** CH, **(b)** OSAVI, **(c)** GNDVI, **(d)** NDRE, **(e)** R475, **(f)** R842, **(g)** SAVI, **(h)** R668, **(i)** R717.

To further investigate the specific influence patterns of individual features on wheat biomass prediction, SHAP dependence plots were generated for the nine most important variables (**Figure 7**). These plots illustrate how the SHAP value (i.e., the contribution of a feature to the model output) changes with the feature value, revealing non-linear relationships and potential interaction effects. In SHAP dependence plots, the magnitude (absolute value) of the SHAP value indicates the strength of the feature’s contribution, while the sign indicates the direction of the contribution (positive or negative).

Canopy height (CH) (**Figure 7a**) exhibited a clear positive linear relationship with its SHAP values. As CH increased from approximately 0.2 to 1.0 (normalized scale), the SHAP value increased almost linearly from negative values (−500) to highly positive values (>400). The large magnitude of negative SHAP values at low CH values indicates that short canopies strongly reduced the predicted biomass, while the large positive SHAP values at high CH values indicate that tall canopies strongly increased the predicted biomass. This strong positive trend is consistent with the biological expectation that plant height is a direct indicator of aboveground biomass accumulation. OSAVI (**Figure 7b**) showed a complex pattern. At lower OSAVI values (0.2–0.4), the SHAP values were positive, while at moderate to high values (0.6–1.0), the SHAP values became predominantly negative. The negative SHAP values at high OSAVI values were of substantial magnitude (approximately −100), indicating that high OSAVI values strongly reduced biomass predictions. This suggests that OSAVI exhibits a non-linear, saturating effect, where excessive greenness or canopy cover may not correspond to further biomass increases and may even lead to overestimation issues in the model. GNDVI (**Figure 7c**) displayed a contrasting bimodal distribution. Low GNDVI values were associated with positive SHAP values, while high GNDVI values corresponded to negative SHAP values with relatively large magnitudes (approximately −100). This indicates that high GNDVI values strongly reduced biomass predictions. This counterintuitive pattern may be explained by the fact that GNDVI is sensitive to chlorophyll content, and at later growth stages, high GNDVI may indicate canopy senescence or changes in leaf structure rather than increased biomass, leading the model to adjust predictions downward. NDRE (**Figure 7d**) exhibited a positive relationship at higher feature values (0.5–1.0), where increasing NDRE values led to higher positive SHAP values. However, at lower NDRE values (0–0.3), the SHAP values were near zero or slightly negative, indicating limited contribution under low chlorophyll conditions. R475 (blue band) (**Figure 7e**) showed a non-linear pattern with two distinct clusters. The SHAP values were positive at lower reflectance values (0–0.4) and became increasingly negative at higher reflectance values (0.6–1.0), with negative SHAP values reaching approximately −50. This indicates that high blue reflectance (low pigment absorption) strongly reduced biomass predictions, which is biologically reasonable as healthy, high-biomass canopies typically exhibit low blue reflectance due to strong pigment absorption. R842 (NIR band) (**Figure 7f**) displayed a positive trend, where increasing NIR reflectance generally led to higher SHAP values. However, the distribution was broader and less linear than CH, indicating interactions with other variables. The positive SHAP values at high R842 values indicate that high NIR reflectance contributed positively to biomass predictions. SAVI (**Figure 7g**) followed a similar pattern to OSAVI, with positive SHAP values at low SAVI values and negative SHAP values at high SAVI values. The magnitude of negative SHAP values at high SAVI values was approximately −25, indicating a moderate negative contribution. R668 (red band) (**Figure 7h**) showed a dip in SHAP values at moderate reflectance levels (0.2–0.5), with slight positive contributions at both low and high extremes. The negative SHAP values at moderate reflectance levels indicate that moderate red reflectance reduced biomass predictions, possibly due to the complex interactions between red reflectance, canopy cover, and chlorophyll absorption. R717 (red edge band) (**Figure 7i**) exhibited a positive linear trend similar to CH, though with a much smaller magnitude of SHAP values (ranging from −25 to +25). The positive relationship indicates that higher red edge reflectance, which is sensitive to leaf internal structure and chlorophyll content, contributed positively to biomass estimation.

Overall, the SHAP dependence plots reveal that while structural variables (CH) show a straightforward positive relationship with biomass, spectral variables often exhibit non-linear, saturating, or threshold-dependent effects. Importantly, large negative SHAP values (e.g., for OSAVI, GNDVI, and R475 at high feature values) indicate that these variables strongly reduced model predictions under certain conditions, which is a meaningful contribution that should not be overlooked. These findings underscore the importance of integrating both structural and spectral information to capture the full complexity of wheat biomass dynamics.

#### 3.5.3 Variable contribution differences across biomass levels

**Figure 8:**
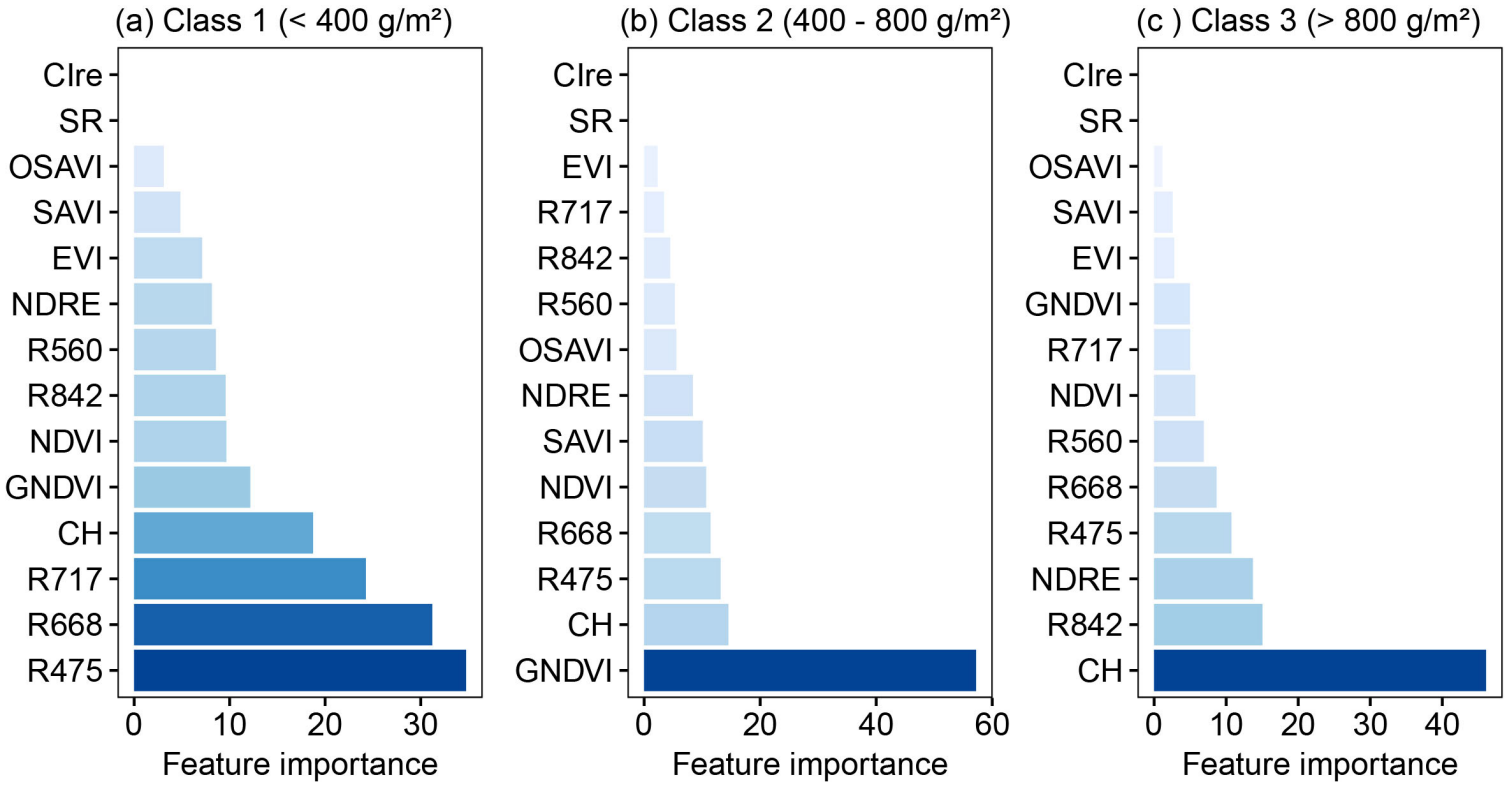
SHAP-based variable contribution differences across wheat biomass levels. **(a)** Class 1 (< 400g/m^2^), **(b)** Class 2 (400 - 800 g/m^2^), **(c)** Class 3 (> 800 g/m^2^).

To further investigate how the importance of input variables varies with wheat biomass level, the dataset was stratified into three biomass classes based on the measured aboveground biomass values: Class 1 (< 400 g/m²), Class 2 (400–800 g/m²), and Class 3 (> 800 g/m²). The biomass levels were classified based on the pooled dataset across all four growth stages. The feature importance for each class, derived from the SHAP analysis of the optimal XGBoost model, is presented in **Figure 8**.

Class 1 (< 400 g/m²) (**Figure 8a**) represents the early growth stages or low-biomass conditions. In this class, the most important variable was the blue reflectance band (R475), with a feature importance score of approximately 32, followed closely by the red reflectance band (R668) with a score of approximately 30. The red edge band (R717) ranked third with a score of approximately 25. Canopy height (CH) ranked fourth with a score of approximately 18, indicating that structural information was less influential in low-biomass conditions. In contrast, vegetation indices such as OSAVI, SAVI, and EVI showed very low importance scores (all below 10), suggesting that spectral reflectance features, particularly in the visible bands, were more effective than vegetation indices for biomass estimation at low biomass levels.

Class 2 (400–800 g/m²) (**Figure 8b**) represents the medium biomass range. A striking shift in variable importance was observed: GNDVI became the dominant variable with a feature importance score of approximately 55, far exceeding all other features. CH ranked second with a score of approximately 15, while the visible reflectance bands (R475, R668, and R560) showed moderate importance with scores ranging from 10 to 15. This dramatic increase in the importance of GNDVI suggests that greenness-related indices are particularly sensitive to biomass variations in the medium range, likely reflecting the active growth phase where canopy chlorophyll content and leaf area index are rapidly increasing.

Class 3 (> 800 g/m²) (**Figure 8c**) represents the high biomass range, typically corresponding to later growth stages such as heading and filling. In this class, canopy height (CH) became the most important variable with a feature importance score of approximately 45. The near-infrared reflectance band (R842) ranked second with a score of approximately 18, followed by NDRE with a score of approximately 12. Notably, the importance of GNDVI decreased dramatically from approximately 55 in Class 2 to approximately 8 in Class 3, indicating that greenness-based vegetation indices saturate at high biomass levels and lose their predictive power. Similarly, the visible reflectance bands (R475, R668, and R560) showed reduced importance compared to Class 1.

Overall, the variable importance patterns varied distinctly across biomass levels. In low-biomass conditions (Class 1), visible reflectance bands (R668 and R475) were the dominant predictors. In medium-biomass conditions (Class 2), GNDVI emerged as the most important variable. In high-biomass conditions (Class 3), canopy height (CH) became the dominant predictor. These findings suggest that the optimal feature set for wheat biomass estimation depends on the target biomass range. For early growth stages with low biomass, spectral reflectance features in the visible bands are most informative. For the active growth phase with medium biomass, greenness-based vegetation indices (e.g., GNDVI) are most effective. For later growth stages with high biomass, structural information (canopy height) provides the most accurate predictions. This highlights the importance of considering biomass level when selecting input variables for machine learning-based biomass estimation models.

## 4 Discussion

### 4.1 Structural information dominates wheat biomass estimation

The results of this study clearly demonstrate that canopy height (CH) is the most important predictor for wheat aboveground biomass estimation, as evidenced by both the global SHAP analysis (**Figure 6**) and the performance comparison of different variable fusion schemes (**Figure 4**). The VI-R-CH scheme, which integrated CH with spectral features, achieved the highest accuracy (R^2^ = 0.919, RMSE = 102.43 g/m²), while the R-CH and VI-CH schemes also outperformed the spectral-only schemes. These findings are consistent with previous studies that have reported canopy height as a key structural parameter for crop biomass estimation. For instance, It was demonstrated that plant height derived from UAV-based structure-from-motion (SfM) photogrammetry was strongly correlated with barley biomass (R^2^ = 0.82) (Bendig et al., 2014). Similarly, it was found that incorporating canopy height information significantly improved the accuracy of wheat biomass estimation compared to using spectral data alone (Liu et al., 2025; Wang et al., 2023).

The dominance of CH can be attributed to the direct biophysical relationship between plant height and aboveground dry matter accumulation. Wheat biomass is primarily composed of stem, leaf, and grain tissues, and the vertical growth of the canopy is a direct reflection of stem elongation and overall plant development. Unlike spectral reflectance, which can be influenced by canopy architecture, soil background, and atmospheric conditions, canopy height provides a more direct and stable indicator of biomass, particularly at later growth stages when spectral indices tend to saturate. This is consistent with our finding that CH became the most important variable in the high-biomass class (> 800 g/m²) (**Figure 8c**).

It should be noted that the observed dominance of CH at high biomass levels was derived from SHAP analysis on the pooled dataset across all growth stages, where Class 3 (> 800 g/m²) included samples from multiple stages. Thus, the pattern reflects cross-stage differences rather than within-stage variation. Furthermore, SHAP-based importance reflects the marginal contribution of a feature within the multi-feature model, which is not equivalent to its bivariate correlation with AGB. Therefore, even when CH exhibits limited within-stage variance, it may still contribute substantially by capturing between-stage differences and interacting with other spectral features. We acknowledge this as a limitation of the pooled-data approach and suggest that future studies employ stage-specific modeling to further disentangle these effects.

It should be noted that this study used canopy height as the sole structural indicator. Other structural metrics, such as vegetation cover fraction and leaf area index (LAI), were not included in our analysis. While canopy height proved to be the most important predictor in our SHAP analysis, these additional structural features may provide complementary information, particularly during early growth stages when height variation is limited. Future studies could explore the integration of multiple structural metrics, such as fractional vegetation cover or LAI, to further enhance biomass estimation accuracy.

### 4.2 Spectral features provide complementary information

Although CH was the most important predictor, spectral features such as R842 (NIR reflectance), GNDVI, and NDRE also contributed significantly to biomass estimation (**Figure 6**). These spectral variables capture the physiological and biochemical properties of the canopy, including chlorophyll content, leaf area index (LAI), and nitrogen status, which are closely linked to photosynthetic capacity and biomass accumulation (Gitelson et al., 2003; Elsayed et al., 2021).

The fusion of spectral and structural data (VI-R-CH) achieved substantially higher accuracy than either spectral-only (VI or R) or CH-only schemes (**Figure 4**). This improvement can be explained by the complementary nature of spectral and structural information. Spectral data are sensitive to changes in leaf pigment concentration and canopy greenness, which are particularly important during the early and active growth stages. In contrast, structural data are more effective for capturing biomass accumulation at later stages when the canopy becomes dense and spectral indices saturate (Banerjee et al., 2020). The combination of these two types of information allows the model to leverage both physiological and morphological characteristics of the crop, leading to more robust and accurate predictions.

The complementary roles of spectral and structural variables were further confirmed by the variable importance analysis across biomass levels (**Figure 8**). In low-biomass conditions (Class 1), spectral reflectance bands (R668 and R475) were the dominant predictors, as the canopy was sparse and spectral signals were less affected by saturation. In medium-biomass conditions (Class 2), GNDVI emerged as the most important variable, reflecting the active growth phase where greenness is a reliable indicator of biomass. In high-biomass conditions (Class 3), CH became the dominant predictor, as spectral indices reached saturation while canopy height continued to increase. These findings are consistent with previous studies that have reported the saturation of vegetation indices at high LAI values (Haboudane et al., 2004; Thenkabail et al., 2000).

### 4.3 Non-linear response patterns of spectral variables

The SHAP dependence plots (**Figure 7**) revealed complex, non-linear relationships between spectral variables and biomass predictions. For example, OSAVI and GNDVI exhibited negative SHAP values at high feature values (**Figures 7b, c**), indicating that excessive greenness or canopy cover reduced biomass predictions. This pattern may be explained by the saturation effect of vegetation indices. When the canopy is fully closed, further increases in greenness or leaf area do not translate into additional biomass accumulation, and the model may penalize extremely high values to avoid overestimation (Thenkabail et al., 2000). Similarly, R475 (blue band) showed negative SHAP values at high reflectance values (**Figure 7e**), which is biologically reasonable as high blue reflectance indicates low chlorophyll absorption, which is associated with lower biomass.

These non-linear response patterns highlight the importance of using non-linear machine learning models such as XGBoost for biomass estimation. Linear models like LASSO, which cannot capture complex interactions and non-linearities, exhibited significantly lower accuracy (R^2^ = 0.803) compared to XGBoost (R^2^ = 0.919) in this study (**Figure 3**). The ability of tree-based ensemble methods to model non-linear relationships and interactions among variables is a key advantage for remote sensing-based crop parameter estimation (Belgiu and Dragut, 2016).

The findings of this study have several practical implications for precision agriculture and UAV-based crop monitoring. First, the strong performance of the full fusion scheme (VI-R-CH) suggests that UAV-based biomass estimation should integrate both spectral and structural data. This can be achieved by equipping UAVs with multispectral cameras and using SfM photogrammetry to generate canopy height models, which is a cost-effective and widely accessible approach (Bendig et al., 2015). Second, the variable importance analysis across biomass levels provides guidance for feature selection tailored to different growth stages. For early-stage biomass estimation (low biomass), priority should be given to visible spectral bands such as R668 and R475. For the active growth phase (medium biomass), greenness-based indices like GNDVI should be emphasized. For late-stage monitoring (high biomass), canopy height should be the primary predictor. This stage-specific strategy can help optimize model performance and reduce the computational burden of including redundant features. Third, the non-linear response patterns observed in this study underscore the importance of using interpretable machine learning methods like SHAP. By providing transparent explanations of model predictions, SHAP analysis enables researchers and practitioners to understand the biophysical basis of the model output, identify potential saturation issues, and refine feature selection strategies (Lundberg and Lee, 2017).

## 5 Conclusions

This study demonstrated that the integration of UAV-based multispectral and structural data, combined with machine learning and SHAP interpretability analysis, provides a robust and interpretable framework for wheat aboveground biomass estimation across multiple growth stages. Among the four machine learning models evaluated, XGBoost achieved the highest estimation accuracy (R² = 0.919, RMSE = 102.43 g/m²), outperforming Random Forest, SVR, and LASSO, highlighting its strong capability in capturing complex non-linear relationships between biomass and multi-source remote sensing features.

The fusion of spectral reflectance, vegetation indices, and canopy height (VI-R-CH) consistently outperformed all other variable combinations, emphasizing the complementary value of structural and spectral information. SHAP-based global and local interpretability analyses revealed that canopy height (CH) was the most influential predictor overall, followed by near-infrared reflectance (R842), GNDVI, and NDRE. More importantly, the contribution of individual variables varied distinctly across biomass levels: visible reflectance bands (R668, R475) dominated in low-biomass conditions (<400 g/m²), GNDVI became the key predictor at medium biomass levels (400–800 g/m²), and canopy height emerged as the dominant variable under high-biomass conditions (>800 g/m²). These findings not only clarify the biophysical mechanisms underlying biomass prediction but also offer practical guidance for stage-specific feature selection.

Overall, this study advances the understanding of how spectral and structural variables contribute to wheat biomass estimation and provides a transparent, interpretable framework for UAV-based crop monitoring. Future research should explore the transferability of this framework to other crops and regions, as well as the integration of time-series data to further improve estimation accuracy and generalizability.

This study demonstrated that the integration of UAV-based multispectral and structural data, combined with machine learning and SHAP interpretability analysis, provides a robust and interpretable framework for wheat aboveground biomass estimation across multiple growth stages. Among the four machine learning models evaluated, XGBoost achieved the highest estimation accuracy (R² = 0.919, RMSE = 102.43 g/m²), outperforming Random Forest, SVR, and LASSO, highlighting its strong capability in capturing complex non-linear relationships between biomass and multi-source remote sensing features. The fusion of spectral reflectance, vegetation indices, and canopy height (VI-R-CH) consistently outperformed all other variable combinations, emphasizing the complementary value of structural and spectral information. SHAP-based global and local interpretability analyses revealed that canopy height (CH) was the most influential predictor overall, followed by near-infrared reflectance (R842), GNDVI, and NDRE.

More importantly, the contribution of individual variables varied distinctly across biomass levels, revealing a clear mechanistic pattern. At low biomass levels (< 400 g/m², corresponding to early growth stages), visible reflectance bands (R668 and R475) dominated the prediction. This is because the sparse canopy allows spectral signals to capture the strong contrast between green vegetation and bare soil, with chlorophyll absorption in the red and blue bands providing sensitive indicators of early photosynthetic activity and biomass accumulation. At medium biomass levels (400–800 g/m², corresponding to active growth phases), GNDVI emerged as the key predictor, as the closing canopy enhanced the sensitivity of greenness-based indices to rapid physiological changes in leaf area and chlorophyll content. At high biomass levels (> 800 g/m², corresponding to later stages), canopy height became the dominant variable. This shift occurs because spectral vegetation indices approach saturation under full canopy cover, whereas canopy height continues to increase with stem elongation and dry matter accumulation, providing a non-saturating structural metric of aboveground biomass. These stage-dependent shifts can be mechanistically understood through the complementary roles of spectral and structural signals: spectral features are most informative when the vegetation–soil contrast is high and the canopy is actively growing, but they lose sensitivity upon canopy closure and spectral saturation; structural features, in contrast, maintain their predictive power throughout the growth cycle and become particularly valuable when spectral signals saturate.

These findings not only clarify the biophysical mechanisms underlying biomass prediction but also offer practical guidance for stage-specific feature selection: visible bands should be prioritized for early-stage estimation, greenness-based indices for the active growth phase, and canopy height for late-stage monitoring. Overall, this study advances the understanding of how spectral and structural variables contribute to wheat biomass estimation and provides a transparent, interpretable framework for UAV-based crop monitoring. Future research should explore the transferability of this framework to other crops and regions, as well as the integration of time-series data to further improve estimation accuracy and generalizability.

## Data Availability

The original contributions presented in the study are included in the article/supplementary material. Further inquiries can be directed to the corresponding author.
